# DNA Methylation: Insights into Human Evolution

**DOI:** 10.1371/journal.pgen.1005661

**Published:** 2015-12-10

**Authors:** Irene Hernando-Herraez, Raquel Garcia-Perez, Andrew J. Sharp, Tomas Marques-Bonet

**Affiliations:** 1 Institute of Evolutionary Biology (UPF-CSIC), PRBB, Barcelona, Spain; 2 Department of Genetics and Genomic Sciences, Icahn School of Medicine at Mount Sinai School, New York, New York, United States of America; 3 Catalan Institution of Research and Advanced Studies (ICREA), Barcelona, Spain; 4 Centro Nacional de Analisis Genomico (CNAG), PCB, Barcelona, Spain; King's College London, UNITED KINGDOM

## Abstract

A fundamental initiative for evolutionary biologists is to understand the molecular basis underlying phenotypic diversity. A long-standing hypothesis states that species-specific traits may be explained by differences in gene regulation rather than differences at the protein level. Over the past few years, evolutionary studies have shifted from mere sequence comparisons to integrative analyses in which gene regulation is key to understanding species evolution. DNA methylation is an important epigenetic modification involved in the regulation of numerous biological processes. Nevertheless, the evolution of the human methylome and the processes driving such changes are poorly understood. Here, we review the close interplay between Cytosine-phosphate-Guanine (CpG) methylation and the underlying genome sequence, as well as its evolutionary impact. We also summarize the latest advances in the field, revisiting the main literature on human and nonhuman primates. We hope to encourage the scientific community to address the many challenges posed by the field of comparative epigenomics.

## Introduction

Methylation of nucleotide bases is the only covalent modification of DNA commonly found across many different taxa. So far, three types have been described: N6-methyladenine (6mA), N4-methylcytosine (4mC), and 5-methylcytosine (5mC). While 6mA and 4mC are restricted to prokaryotes and certain eukaryotes [1–3], 5mC is the predominant epigenetic modification in eukaryotic DNA [4]. In mammals, 5mC mainly occurs in the context of Cytosine-phosphate-Guanine (CpG) dinucleotides [5].

The distribution of CpG methylation is uneven across mammalian genomes. While the majority of CpGs (~60%–80%) are methylated [5], regions of densely clustered CpGs, known as CpG islands (CGIs), are often devoid of methylation [6]. Many CGIs are found in the vicinity of gene promoters, with approximately two-thirds of genes having a CGI at their promoter. Methylation of promoter CGIs provokes long-term transcriptional repression of the associated genes [5,7]. Classical examples include X chromosome inactivation [8] and genomic imprinting [9]. In contrast, the functional impact of DNA methylation outside gene promoters is not so well understood. Gene body methylation has been reported to be involved in alternative splicing [10], and methylation of transposable elements is involved in the suppression of retrotransposition [11]. Finally, certain unmethylated domains in CpG-poor regions have been shown to coincide with distal regulatory elements, such as active enhancers, and usually co-locate with other epigenetic marks, such as histone modifications [12–15]. Together, these findings suggest that DNA methylation patterns are complex and highly dependent on the genomic context [16].

Non-CpG methylation, the methylation of cytosines followed by a base other than guanine, has also been reported. Indeed, non-CpG methylation is abundant in plants [17,18] and recently has also been identified in some mammalian cell types [12,18–20]. Furthermore, additional chemical modifications arisen from the oxidative conversion of 5mC have also been described [21–24]. The significance of these epigenetic marks in the mammalian genome is currently poorly understood, so our focus is on methylation that occurs in CpG dinucleotides

Despite being a key regulator of genomic function, the role of DNA methylation in species evolution is only beginning to be explored. More than four decades ago, it was first proposed that regulatory changes could lead to species-specific adaptations as well as phenotypic variability [25]; however, technical limitations at that time prevented this hypothesis from being tested. Within the past decade, the development of high-throughput genomic technologies has allowed the study of species evolution from a molecular perspective. Taking advantage of these technologies, recent studies have provided the first insights into the evolution of the epigenome [26]. Some of the key questions in the field are starting to be addressed: Is regulatory evolution coupled with sequence evolution? To what extent do regulatory changes affect transcription? What are the evolutionary landmarks of DNA methylation? In this review, we survey the incipient field of evolutionary epigenomics, focusing on CpG methylation. We discuss the current state of the field and conclude by suggesting future research avenues.

## The Interplay between the Genome and the Methylome

There is a close interplay between DNA methylation and the underlying nucleotide sequence. The mutagenic nature of 5mC is one of the primary players in this crosstalk, as methylcytosines are prone to spontaneous deamination to thymine, resulting in a CpG-to-Thymine-phosphate-Guanine (TpG) mutation rate that is ~10-fold higher than for other dinucleotides [27]. This is reflected in the nucleotide divergence between primates: While the average human-chimpanzee divergence is ~1% across the genome, at CpG sites it increases to ~15% [28]. Furthermore, heavily methylated, CpG-rich, subtelomeric regions exhibit a high rate of deamination, which is balanced by the rapid gain of guanines and cytosines, commonly attributed to biased gene conversion [29]. Nonetheless, in a proportion (~15%) of these genomic regions, the loss of CpG dinucleotides is not compensated [29]. Indeed, high deamination rates over evolutionary time are thought to be the cause of the overall depletion of CpGs that is characteristic of mammalian genomes.

In contrast to the mutagenic effect of 5mC, many genomic regions with high densities of CpGs are constitutively unmethylated. Recent studies suggest that this CpG richness can be solely accounted for by low CpG deamination rates, rather than being a byproduct of purifying selection [29]. About 80% of these regions are located close to (<10 Kb) annotated transcription start sites (TSSs), and meet the classic definition of CpG islands [30]. Therefore, the methylation state plays an important role in the dynamics of CpG dinucleotides.

On the other hand, several lines of evidence indicate that the genome harbors the information necessary to establish local DNA methylation patterns. How this genomic information is encoded and read is still not fully understood, but recent work is beginning to shed light on the regulatory mechanisms. Fragment-insertion experiments have demonstrated that both a high Guanine-phosphate-Cytosine (GC) content and a high density of CpG dinucleotides are necessary and sufficient requirements to induce an unmethylated state [31,32]. From a mechanistic standpoint, it is unclear how CpG and GC content richness drive the unmethylated state. Proteins that specifically recognize unmethylated CpGs via CXXC domains could prevent DNA methylation [33–35]. Other hypotheses posit the existence of proteins that recruit DNA methyltransferases through the recognition of Adenosine-phosphate-Thymine (AT)-rich regions [31].

**Fig 1:**
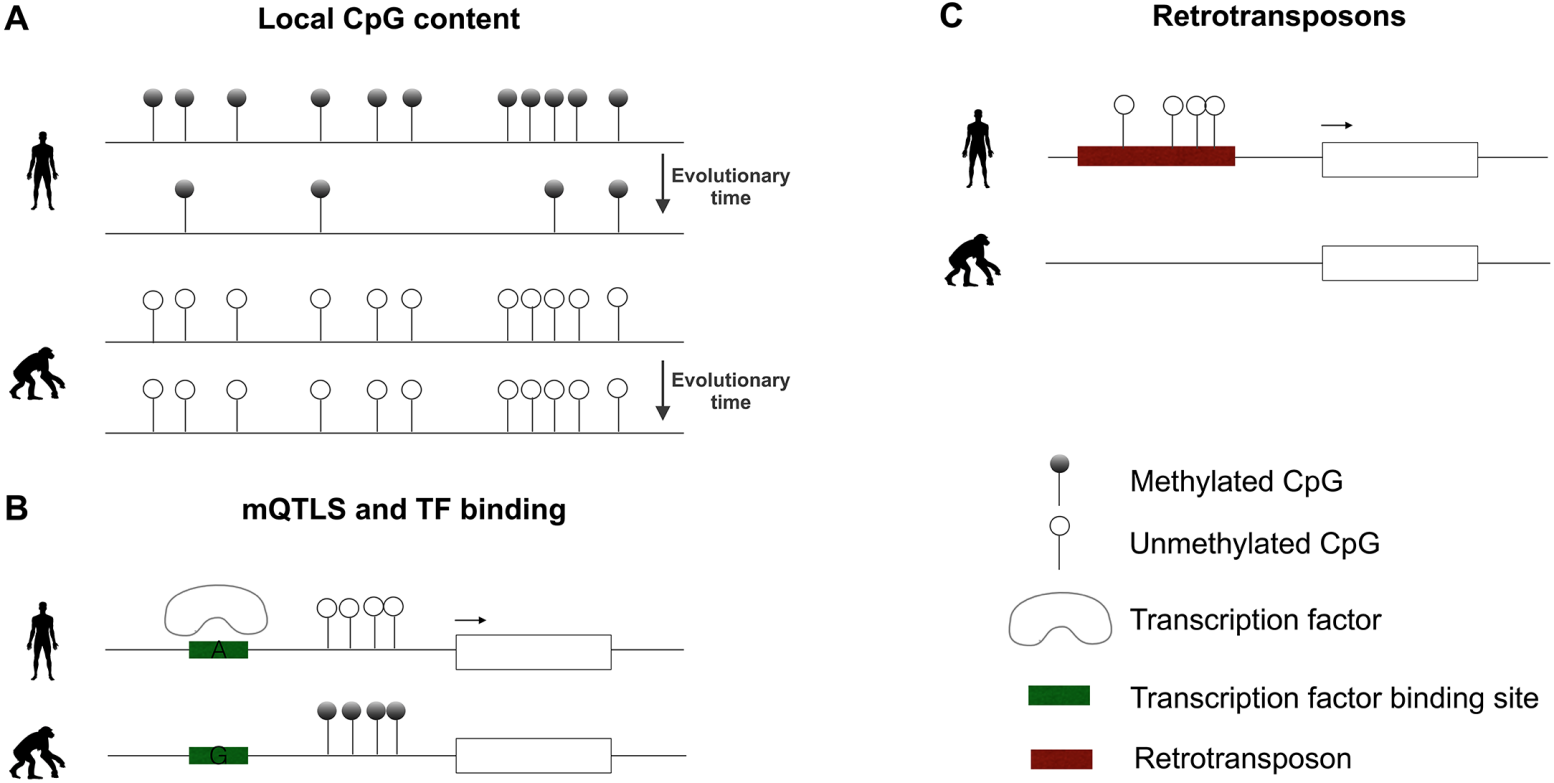
The interplay between the genome and the methylome. A) Methylated cytosines tend to deaminate over evolutionary time and, thus, the methylation state of cytosines in different species influences the evolution of the underlying genome sequence. B) Species-specific nucleotide changes that disrupt transcription factor (TF) binding sites can alter the methylation state of nearby CpG dinucleotides and, as a consequence, establish species-specific differentially methylated regions (DMRs). C) The insertion of transposable elements in a particular lineage, along with the accumulation of nucleotide changes, can lead to the emergence of novel CpG dinucleotides, creating species-specific regulatory regions.

Therefore, evolutionary changes in CpG dinucleotides and GC content could contribute to alter the methylation landscape of a species (Fig 1A). With this idea in mind, regions with high CpG density that are present exclusively in the human lineage have been identified and termed “CpG beacons” [36]. Although their methylation state has not been studied, these regions are good candidates to show differential methylation between species. Interestingly, human-specific CpG beacons were found to be enriched for genes related to cognition and behavior, including the well-characterized *HAR1A* gene, which plays a crucial role in cortical development [37].

Different mechanisms govern methylation patterning at CpG-poor regions. Some compelling studies have revealed that methylation patterns at such regions are likely driven by the binding of transcription factors (TFs), as the presence of specific motifs suffices to create unmethylated domains [13,32,38,39]. Importantly, this mechanism likely plays a crucial role during cell differentiation [40]. Further evidence results from the analysis of methylation patterns after recent duplication events in the human genome. Though most duplicated segments share conserved methylation states, duplication pairs with discordant methylation are associated with nucleotide changes at particular binding motifs [41].

Evolutionary studies in human populations have identified genetic variants that associate with methylation levels at nearby CpGs, termed methylation quantitative trait loci (mQTL) [42–45]. Consistent with the role of TFs in determining methylation patterns, genetic variants that disrupt TF binding sites are more frequently associated with changes in methylation (Fig 1B). Furthermore, TF binding sites within regions that show differential methylation in the human lineage compared to great apes show an increase of human-specific mutations [46]. Altogether, these findings suggest that evolutionary changes in TF binding motifs contribute to shaping the methylome between species.

Finally, it has also been suggested that retrotransposition events coupled with gradual nucleotide changes could lead to the accumulation of novel CpG sites [47]. These regions would provide the necessary grounds for the emergence of new regulatory regions (Fig 1C). Indeed, several reports suggest that imprinting at certain genes could have arisen as a byproduct of DNA methylation silencing of retrotransposons. One such case is the *RB1* imprinted gene, which results from the differential methylation of a processed pseudogene [48].

The emerging picture is that the joint actions of all the above-discussed mechanisms throughout the evolutionary scale have modeled the architecture of the methylome (Fig 1).

## Comparative Epigenomics

Comparative epigenomics, the interspecies comparison of DNA and histone modifications, is a promising research field that can enlighten our understanding of mammalian epigenome evolution [49]. Furthermore, together with TF footprints and chromatin accessibility maps, it can be used as a tool to map regulatory elements [50,51]. Evolutionary studies have shown that 90% of unmethylated regions that are associated with gene promoters are shared across distant vertebrates, from zebrafish to humans, indicating that the unmethylated state at gene promoters is a deeply conserved epigenetic feature [52]. Histone modifications are also conserved at promoters, in sharp contrast to enhancers, whose evolution is much more rapid and less constrained [50].

Importantly, co-localization of different epigenetic marks, including DNA methylation, several histone modifications, and H2A.Z, has been used to study the evolution of the epigenome, the transcriptome, and the genome in humans, mice, and pigs [49]. This study revealed several important points: (i) the co-occurrence of different epigenetic marks is conserved across species, (ii) histone modifications are predictive of gene expression levels in all three species and can explain more than 50% of the variance in gene expression [49,53,54], and (iii) interspecies epigenetic variation often occurs in neutrally evolving regions, leading to the suggestion that the epigenome might act as a buffer by masking genetic changes from immediate phenotypic changes [49]. Such deep comparative epigenetic analyses are extremely valuable and provide the groundwork to characterize general features of the species epigenomes [49,50,55]. Yet, only when we identify the differences that separate us from our closest nonhuman relatives do we get closer to understanding the uniqueness of our species.

## Human and Nonhuman Primates

Though methylomes are overall quite well-conserved between primate species [56,57], several studies have now identified hundreds of species-specific differentially methylated regions (sDMRs) (Table 1). Early studies focused exclusively on methylation levels at gene promoters and CGIs [58,59], providing some interesting examples, including sDMRs in the brain and associated with known disease genes [60–62]. On the premise that changes in gene expression patterns are major contributors to evolutionary innovation [63–66], several studies have also attempted to link expression differences with underlying epigenetic changes at regulatory regions. Early studies using methylation arrays with limited coverage suggested that differential promoter methylation underlies only ~12%–18% of observed gene expression differences between human and chimpanzee [67]. However, other studies focusing on histone modifications indicate that a larger proportion (~40%) of differentially expressed genes can be accounted for by interspecies epigenetic differences [49,53,54]. More recent genome-wide DNA methylation studies have shown that most sDMRs actually locate distal TSS, and that these sites are also enriched in particular histone modifications [46]. This finding highlights the importance of epigenetic modifications at distal regulatory elements, as well as the close interplay between different epigenetic marks. Of note, it has been observed that regions of methylation divergence often coincide not only with regulatory elements of genes functionally related to the tissue being studied but also with genes with functions specific to other tissues and developmental time points [56,62,67]. Though it is unclear how to explain the latter observation, it could be due to vestigial regulatory elements that were functional during development, and their methylation state has remained unaltered in adult tissues [68]. This is an engaging interpretation, since many human-specific morphological features emerge during early development. Indeed, developmental stages are crucial for the acquisition of human-specific traits, and species-specific differences in histone modifications have also been reported in genes involved in developmental processes. Recent studies have shown that many genes that have gained H3K27ac marks since the human-macaque split have important roles in limb development or the acquisition of human-specific traits [69]. Similarly, thousands of promoters and enhancers associated with genes crucial in cortical development were reported to display human-specific gains both in H3K27ac and H3K4me2 [70].

**Table 1:** Comparative studies of DNA methylation patterns in primates.

Reference	Species	Methodology	Tissue	Highlights
Wang, J. (2012)	Human, Macaque	MeDIP-chip and SEQUENOM MassARRAY	Prefrontal cortex	>100 differentially methylated regions; Validated DMRs associated with genes with neural functions and with schizophrenia and Alzheimer's disease
Pai, A. (2011)	Human, Chimpanzee	Illumina 27K array	Liver, heart, and kidney	14.5% of promoter CpG sites are differentially methylated between tissues; 8.6% of promoter CpG sites are differentially methylated between species; Interspecies differences in promoter methylation underlie 12%–18% of gene expression differences
Molaro, A. (2011)	Human, Chimpanzee	Whole-genome bisulfite sequence	Sperm	70% of genes are hypomethylated in both chimpanzee and human sperm; 6% and 35% of orthologous SVAs had a methylation level below 50% in chimpanzee and human sperm, respectively
Martin, D.I.K. (2011)	Human, Chimpanzee, Orangutan	MethylSeq	Neutrophils	10% of CpG islands-like regions present different methylation states between chimpanzees and humans; Regions with differential methylation might have diverged in gene regulatory function
Fukuda, K. (2013)	Human, Chimpanzee	MeDIP-chip (chromosomes 21 and 22)	Peripheral blood leukocytes	16 sDMRs between chimpanzees and humans in chromosomes 21 and 22; Genetic changes underlying these differences in methylation include gain/loss of CTCF-binding sites and changes in CpG density
Hernando-Herraez, I. (2013)	Human, Chimpanzee, Bonobo, Gorilla, Orangutan	Illumina 450K array	Peripheral blood	~9% of the assayed CpG sites showed significant methylation differences between chimpanzees and humans; 184 genes perfectly conserved at protein level show significant epigenetic differences between chimpanzees and humans
Hernando-Herraez, I. (2015)	Human, Chimpanzee, Gorilla, Orangutan	Whole-genome bisulfite sequence	Peripheral blood	72% of the hypomethylated regions (HMRs) were shared among all four species; 42.6% of HMRs were on human CpG islands; 52.6% of HMRs were on human CpG shores
Gokhman, D. (2014)	Neandertal, Denisovan	Deamination rate as a proxy for DNA methylation	Femur, costae, and tibia bones	>2,000 DMRs between archaic and present-day humans; Substantial changes in methylation in the *HOXD* cluster
Fraser, H. B (2012)	Human	Illumina 27K array	Lymphoblastoid cell lines	21.4% of CpG sites differed in methylation between populations; 5.4% of these CpG sites were strongly associated with local SNPs
Heyn, H. 2013	Human	Illumina 450K array	Lymphoblastoid cell lines	439 population-specific differentially methylated CpG sites (pop-CpG); Significantly decreased gene expression associated to promoter hypermethylation in 12.9% (13 out of 101) of pop-CpG; Significantly increased gene expression associated to gene body methylation in 23.9% (27 out of 113) of pop-CpG

In sum, from primate epigenetic comparative studies, we have started to gain insight into the molecular mechanisms through which evolutionary novelties may have arisen in the human lineage. These studies suggest that epigenetic modifications in regulatory elements could have altered expression patterns of key genes, leading to evolutionary adaptations.

## Humans and Extinct Hominids

Genome sequencing of extinct hominids has provided a better understanding of the population history and genome evolution of our species [71,72]. However, additional studies have been limited due to the tiny amounts and degraded nature of DNA that can be extracted from ancient bones. Recently, a novel method was developed to infer DNA methylation patterns from the genome sequence of Neandertals and Denisovans, opening the possibility of studying for the very first time the methylome of extinct species [73]. This method is based on the different spontaneous deamination rates of methylated and unmethylated cytosines, and takes advantage of the characteristic CpG-to-TpG substitution pattern of methylated cytosines that accumulate over thousands of years of chemical degradation. Using this approach, over a thousand DMRs among humans, Neandertals, and Denisovans were identified. Of particular note, one human-specific DMR was located in the *HOXD* cluster, a key regulator of limb development [74]. Whereas the *HOXD9* promoter and *HOXD10* gene body are hypomethylated in humans, both archaic species were hypermethylated at this locus, while the gene body of *HOXD9* was also hypermethylated in the Denisovan genome. As a result, the authors postulated that this differential methylation in the *HOXD* cluster could account for some of the anatomical differences between archaic and present-day humans [73]. However, these results are based on the analysis of the genome of just one individual of each species, and thus it is possible that the observed differences could simply represent epigenetic polymorphisms. Furthermore, to determine if these DMRs truly represent human-specific changes, the ancestral methylation state is required. To date, this has not been feasible due to the lack of primate DNA methylation data from bone tissues.

Few studies have addressed DNA methylation patterns across human populations [44,75]. Fraser et al. [75] reported differences in DNA methylation near TSS between European and African populations, although these changes were relatively small in magnitude and did not show any apparent correlation with gene expression levels. Importantly, while over half of these differences could be accounted for by common cis-linked genetic variants, they also acknowledge that more complex genetic interactions and environmental factors may contribute to DNA methylation variation. Heyn et al. [44] reported that methylation patterns characteristic of three distinct human populations (Caucasian, African-American, and Han Chinese) were able to recapitulate the demographic history of each group. Population-specific DMRs, besides being associated with several histone modifications and TF binding sites, were found to occur in genes related to susceptibilities to different diseases and xenobiotic response factors. These findings suggest that certain methylation changes might be due to local selective pressures, such as geographic differences in pathogens or other environmental factors.

There is now accumulating evidence indicating the influence of different environmental factors on methylation patterns [76]. Although it is unclear the extent to which this phenomenon occurs, it is reasonable to assume that different environments could lead to epigenetic divergence. Given that it is not possible to control the environment in most studies involving non-model organisms, the results need to be carefully interpreted: some of the observed differences between species or populations might be due to environmental factors and not the result of heritable regulatory changes. Further research is required to understand the crosstalk between the genome, the epigenome, and the environment.

## Inheritance of Epigenetic Variation

DNA methylation patterns can be influenced by stochastic events or environmental factors creating a source of epigenetic variability [77,78]. Whether this variation could be passed on to future generations has been the subject of intense research [79,80]. A major barrier for its transmission is the robust reprogramming that resets almost all epigenetic marks both in the germline and the zygote [81]. In plants, however, it is a relatively common phenomenon, mainly attributed to the limited reprogramming that occurs in the germline [82–84]. On the contrary, the inheritance of epigenetic changes in mammals is extremely rare. Nonetheless, if some loci were to escape the DNA demethylation process, they would be good candidates for experiencing epigenetic inheritance. Recent genome-wide studies assessing mouse and human germ cell reprogramming have reported a few genes that avoided this erasure [85–87]. Remarkably, some of these genes were related to metabolic and neurological disorders [87]. Other studies have also described cases in which transgenerational epigenetic inheritance might have occurred [88–91]. These epigenetic alterations are unstable over time, and the phenotypic effects disappear in a few generations. Moreover, changes in the genetic sequence are often disregarded as sources of observed heritability.

The notion of transgenerational epigenetic inheritance is very appealing, particularly when suggested that it could respond to environmental challenges [92]. In such situations, it has been proposed that the persistence of epigenetic changes across generations could be considered adaptive and, therefore, it might impact fitness and influence species evolution [93]. Despite this provocative idea, to date there is no solid evidence to substantiate this claim, particularly in mammals, and experimental attempts have failed to prove an adaptive role of epigenetic variation [79].

## Future Directions

Our understanding of DNA methylation and evolution has substantially grown over the past years. Nonetheless, the field of evolutionary epigenomics is still in its infancy, and several steps are required to complete an accurate and detailed picture of the human epigenome.

Despite remarkable achievements accomplished through the parallel survey of genetic, epigenetic, and transcriptional information [40,94,95], a comprehensive evolutionary perspective is required to elucidate the complexity of regulatory mechanisms and to assess the significance of epigenetic changes. Such integrative studies will also be key to interpreting noncoding variation. Besides, many comparative studies disregard the spatial and temporal dynamics of DNA methylation and have exclusively focused on adult organs. Notably, studying embryonic developmental stages is crucial to understanding species evolution, since many of the phenotypically relevant changes occur at these stages. The usage of bulk tissue samples, composed by multiple and variable proportions of cell types, is also problematic. However, the recently developed single-cell bisulfite sequencing will likely overcome this limitation [96].

Future studies also need to determine which genomic regions are more susceptible to experiencing epigenetic changes induced by environmental factors, since such regions could represent confounding factors when studying interspecies epigenetic differences. Studies of methylation patterns in populations with similar genetic backgrounds but different environments would be a good starting point. On the other hand, non-CpG methylation as well as 5mC derivatives have only been recently reported, and they have not been studied in an evolutionary context.

Finally, the major challenge for the next years is to move beyond mere comparative descriptions and offer insight into phenotypic consequences. To that end, experimental assays are required. In this regard, humanized mice have proved useful resources [97,98]. However, they might not be suitable for interpreting certain phenotypes; perhaps induced pluripotent stem cells (iPSCs) could fill this gap [99]. iPSCs can be differentiated to several cell types, providing a system in which to investigate the phenotypic effects of interspecies differences. We foresee an exciting decade for the field.
